# Außerhäusliche Mobilität von Personen ab 75 Jahren im ländlichen Raum. Ergebnisse aus einer GPS-Studie

**DOI:** 10.1007/s00103-024-03917-2

**Published:** 2024-07-01

**Authors:** Christine Haeger, Sandra A. Mümken, Robert P. Spang, Max Brauer, Jan-Niklas Voigt-Antons, Paul Gellert

**Affiliations:** 1 Institut für Medizinische Soziologie und Rehabilitationswissenschaft, corporate member of Freie Universität Berlin and Humboldt Universität zu Berlin, Charité – Universitätsmedizin Berlin, Charitéplatz 1, 10117 Berlin, Deutschland; 2 Quality Usability Lab, Technische Universität Berlin, Berlin, Deutschland; 3 Center of Geographic Analyses, Harvard University, Cambridge, MA USA; 4 Hochschule Hamm-Lippstadt, Hamm, Deutschland

**Keywords:** Ländlicher Raum, Außerhäusliche Mobilität, Ältere Menschen, Inanspruchnahme, Versorgung, Rural areas, Out-of-home mobility, Community-dwelling older adults, Utilization of health care, Outpatient care

## Abstract

**Hintergrund:**

Außerhäusliche Mobilität, definiert als aktives und passives Bewegen durch außerhäusliche Umwelten, ist eine Ressource für Autonomie, Lebensqualität und Selbstverwirklichung im Alter. Beeinflusst wird diese multifaktoriell, was bisher vor allem im urbanen Raum untersucht wurde. Ziel der Studie ist es, assoziierte Faktoren in einer ländlichen Studienpopulation ab 75 Jahren zu untersuchen.

**Methoden:**

Baseline-Daten der MOBILE-Studie von 212 Personen ab 75 Jahren erhoben zwischen Juni 2021 und Oktober 2022 gingen in die Analysen ein. Außerhäusliche Mobilität (GPS-basiert an 7 aufeinanderfolgenden Tagen) wurde zeitlich als Time out of Home (TOH) sowie räumlich als Convex Hull (CHull) gemessen. Gemischte Modelle berücksichtigten neben ambulanten Versorgungsparametern persönliche, soziale und umweltbezogene Faktoren sowie Kovariaten wie Alter und Geschlecht.

**Ergebnisse:**

Die Teilnehmenden der MOBILE-Studie (M_Alter_ 81,5, SD: 4,1, davon 56,1 % weiblich) zeigten eine tägliche außerhäusliche Mobilität von M_TOH_: 319,3 min; SD: 196,3 und M_CHull_: 41,3; SD: 132,8. Signifikante Assoziationen wurden für Alter (TOH: ß = −0,039; *p* < 0,001), soziales Netzwerk (TOH: ß = 0,123; *p* < 0,001), Zusammenleben (CHull: ß = 0,689; *p* = 0,035), Gesundheitskompetenz (CHull: ß = 0,077; *p* = 0,008), Gehwegqualität (ß = 0,366; *p* = 0,003), Grünflächenanteil (TOH: ß = 0,005; *p* = 0,047), ambulante Versorgungsinanspruchnahme (TOH: ß = −0,637; *p* < 0,001, CHull: ß = 1,532; *p* = 0,025) und aktives Autofahren (TOH: ß = −0,361; *p* = 0,004) gefunden.

**Diskussion:**

Bereits bekannte multifaktorielle Assoziationen mit objektiv gemessener außerhäuslicher Mobilität konnten im ländlichen Raum bestätigt werden. Neuartig und für Forschung und Praxis relevant ist der signifikante Zusammenhang außerhäuslicher Mobilität mit der Versorgungsinanspruchnahme.

## Abstract (en)

**Background:**

Out-of-home mobility, defined as active and passive movement through external environments, is a resource for autonomy, quality of life, and self-realization in older age. Various factors influence out-of-home mobility, primarily studied in urban settings. The study aims to examine associated factors in a study population aged 75 and above in rural areas.

**Methods:**

Baseline data from the MOBILE trial involving 212 participants aged 75 and above and collected between June 2021 and October 2022 were analyzed. Out-of-home mobility was measured temporally as time out of home (TOH) and spatially as convex hull (CHull) using GPS over seven days. Mixed models considered outpatient care parameters as well as personal, social, and environmental factors along with covariates such as age and gender.

**Results:**

Participants in the MOBILE study (average age 81.5; SD: 4.1; 56.1% female) exhibited average out-of-home mobility of TOH: 319.3 min (SD: 196.3) and CHull: 41.3 (SD: 132.8). Significant associations were found for age (TOH: ß = −0.039, *p* < 0.001), social network (TOH: ß = 0.123, *p* < 0.001), living arrangement (CHull: ß = 0.689, *p* = 0.035), health literacy (CHull: ß = 0.077, *p* = 0.008), sidewalk quality (ß = 0.366, *p* = 0.003), green space ratio (TOH: ß = 0.005, *p* = 0.047), outpatient care utilization (TOH: ß = −0.637, *p* < 0.001, CHull: ß = 1.532; *p* = 0.025), and active driving (TOH: ß = −0.361, *p* = 0.004).

**Discussion:**

Previously known multifactorial associations related to objectively measured out-of-home mobility in old age could be confirmed in rural areas. Novel and relevant for research and practice is the significant correlation between out-of-home mobility and outpatient care utilization.

## Hintergrund

Mobilität im Alter ermöglicht Autonomie, Lebensqualität und Selbstverwirklichung und stellt in der Wahrnehmung älterer Menschen einen Teil der eigenen Identität dar [1]. Dabei kann der Begriff der Mobilität sehr unterschiedlich definiert werden, unter anderem als gemessene aktive Bewegungsfähigkeit, als Fähigkeit zur Durchführung von Alltagsaktivitäten oder durch die Möglichkeiten zur Teilnahme am Straßenverkehr [2]. Dieser Beitrag fokussiert sich auf die Mobilität außerhalb des eigenen Zuhauses und nimmt die Definition des Life-Space-Mobilitätskonzeptes als aktive oder passive Bewegung durch außerhäusliche Umwelten zur Grundlage [3]. Diese Definition schließt sämtliche Fortbewegungsarten ein, die sowohl aktiv (beispielsweise Zufußgehen oder Fahrradfahren) oder passiv (beispielsweise Automobilität oder Nutzung des Öffentlichen Personennahverkehrs – ÖPNV) sein können.

In Mobilitätsstudien wird zunehmend eine objektive Erfassung außerhäuslicher Mobilität mittels Global Positioning System (GPS) verwendet, die im Vergleich zur subjektiven Erfassung mittels Fragebogen robuster gegenüber Überschätzung und Erinnerungsverzerrung ist [4]. Als Faktoren, die mit außerhäuslicher Mobilität assoziiert sind, wird sowohl in der theoretischen Literatur als auch in Studien eine Reihe von persönlichen, sozialen und umweltbezogenen Faktoren beschrieben [1, 5]. Hierzu zählen unter anderem die körperliche Gesundheit und körperliche Aktivität [6, 7], kognitive Funktionsfähigkeit [8], soziale Partizipation [9], Fahrtauglichkeit [5], Gesundheitskompetenz [10]. Weiterhin – aber bisher noch wenig untersucht – können Versorgungsparameter wie die Inanspruchnahme ambulanter Versorgungseinrichtungen einen assoziierten Faktor darstellen [11]. Doch insbesondere der Zusammenhang außerhäuslicher Mobilität älterer Menschen mit Faktoren der physischen und sozialen Umwelt ist komplex und nicht eindeutig geklärt [10].

Außerhäusliche Umwelten sind unter anderem geprägt durch Bebauung, Anteil an Natur- und Grünflächen, physische Beschaffenheit (z. B. die Kreuzungsdichte der Fußwege als Maß der Fußgängerfreundlichkeit oder die Straßenqualität) sowie die vorhandene Infrastruktur (z. B. die ÖPNV-Anbindung oder die Anzahl von Einkaufsmöglichkeiten; [12, 13]). Zudem können sich diese Faktoren stark zwischen städtischen und ländlichen Räumen unterscheiden und als förderliche oder hinderliche Faktoren für die außerhäusliche Mobilität auftreten [14]. In ländlichen Regionen zeigt sich, dass oftmals weite Wege notwendig sind, um Alltagsaktivitäten nachzugehen oder gesundheitliche Versorgungsleistungen wahrzunehmen. Gerade die Inanspruchnahme gesundheitlicher Versorgungsleistungen auf dem Land kann zukünftig kritischer werden, da die Bereitschaft von Ärztinnen und Ärzten, Hausbesuche anzubieten, weiter sinkt [15] und ältere Personen unter anderem auf die ÖPNV-Anbindung, die eigene Fahrtauglichkeit sowie auf soziale Unterstützungsmöglichkeiten angewiesen sind [16].

In Bezug auf die gesundheitliche Versorgung ist eine eingeschränkte außerhäusliche Mobilität unter anderem mit einer erhöhten Inanspruchnahme von Notfallleistungen [17], Pflegeheimzuweisungen [18] und körperlichen Einschränkungen [19] assoziiert. Zusammenhänge dieser genannten Faktoren sowie der ambulanten außerhäuslichen Versorgung mit objektiv gemessener außerhäuslicher Mobilität sind insbesondere im ländlichen Raum wenig untersucht [11]. Dabei bieten veränderliche Faktoren der physisch-sozialen Umwelt sowie die Förderung persönlicher Kompetenzen gute Möglichkeiten für die Entwicklung niedrigschwelliger Angebote zur Förderung außerhäuslicher Mobilität und Gesundheit älterer Menschen [5].

Das Ziel der vorliegenden Studie ist es, zu untersuchen, inwieweit im urbanen Raum bereits etablierte Zusammenhänge von persönlichen, sozialen und umweltbezogenen Mobilitätsfaktoren auch mit der objektiv per GPS gemessenen außerhäuslichen Mobilität von Personen ab 75 Jahren im ländlichen Raum gefunden werden können und welche Zusammenhänge sich zwischen objektiv gemessener außerhäuslicher Mobilität und der ambulanten Gesundheitsversorgung älterer Menschen im ländlichen Raum ergeben. Dazu werden die folgenden Hypothesen untersucht:

1. Als persönliche Faktoren sind eine gute körperliche und psychische Gesundheit sowie persönliche Kompetenzen (Bildung, Gesundheitskompetenz, aktives Autofahren) positiv mit außerhäuslicher Mobilität assoziiert.
2. Als Faktoren der physischen Umwelt sind Kriterien der Walkability (Gehwegqualität, Kreuzungsdichte der Fußgängerwege), eine attraktive Umwelt (Anteil an Grünflächen) und die ÖPNV-Anbindung positiv mit außerhäuslicher Mobilität assoziiert. Die soziale Umwelt wird durch die Zufriedenheit mit dem sozialen Netzwerk abgebildet und ist ebenfalls positiv mit außerhäuslicher Mobilität assoziiert.
3. Die Versorgung ist durch Zugänglichkeit (subjektive Erreichbarkeit, Anzahl an Gesundheitseinrichtung) und die Inanspruchnahme ambulanter Gesundheitseinrichtungen positiv mit außerhäuslicher Mobilität assoziiert.

## Methoden

### Studienpopulation, Studiendesign und Setting

Die vorliegende Arbeit basiert auf Baseline-Daten der MOBILE-Studie, einer randomisiert kontrollierten Studie mit dem Ziel, die Mobilität von Menschen ab 75 Jahren zu fördern. Die Studie wurde im Landkreis Havelland (Brandenburg) durchgeführt und die Datenerhebung der Baseline-Daten erfolgte zwischen Juni 2021 und Oktober 2022. Der Landkreis Havelland liegt westlich von Berlin und ist als dünn besiedelter ländlicher Kreis gemäß den siedlungsstrukturellen Kreistypen des Bundesinstituts für Bau‑, Stadt- und Raumforschung (BBSR) kategorisiert [20].

Die Studie wurde vom Bundesministerium für Bildung und Forschung (BMBF; ID: 01GY1803) gefördert und im Deutschen Register Klinischer Studien vorregistriert (DRKS00025230, 04.05.2021). Einzelheiten zum Studiendesign wurden im Studienprotokoll [21] beschrieben.

Die Datenerhebung erfolgte in Hausbesuchen mittels CAPI (Computer Assisted Personal Interview). Die Baseline-Erhebung bestand aus einem Fragebogen mit validierten Instrumenten, physischen Messungen sowie einer anschließenden 7‑tägigen Mobilitätsmessung mittels GPS und Bewegungstagebuch. Für die GPS-Messung wurde ein handelsübliches Studien-Smartphone mit der von der Technischen Universität Berlin entwickelten Applikation „GPS.Rec.2.0“ verwendet [22]. Die App zeichnete die GPS-Daten offline und datenschutzkonform auf und eine Übertragung der GPS-Daten für die Auswertung fand erst nach Abschluss der Datenerhebung statt. Details zur technischen Umsetzung finden sich in der Veröffentlichung von Spang et al. [23]. Für die Teilnahme an der Studie galten die folgenden Einschlusskriterien: Alter ab 75 Jahren, Einwilligungsfähigkeit der Teilnehmenden, ausreichende Seh- und Hörfähigkeit zum Verständnis der Fragebögen, ausreichende Bewegungsfähigkeit zur außerhäuslichen Mobilität, Wohnsitz im Landkreis Havelland. Die Ausschlusskriterien waren wie folgt definiert: starke kognitive Einschränkungen, Personen in stationären Pflegeeinrichtungen, schwere psychische oder emotionale Beeinträchtigungen, Vorliegen einer akuten Notfallsituation und schwere mobilitätseinschränkende Ereignisse in den vergangenen 4 Wochen.

### Erfassung der Variablen

**Abb. 1:**
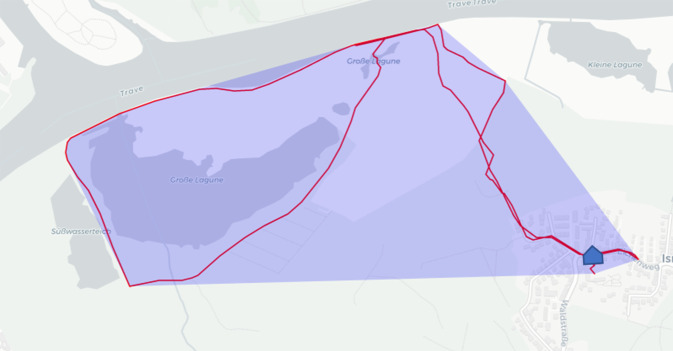
Veranschaulichung der GPS-Variable CHull (konvexe Hülle) als Darstellung der räumlichen Facette der außerhäuslichen Mobilität. Der *blau* markierte Bereich zeigt die CHull eines Tages (Quelle: eigene Abbildung)

Neben den soziodemografischen Angaben Alter, Geschlecht (binär; Frauen = 1, Männer = 0), Bildungsgrad (operationalisiert nach International Standard Classification of Education ISCED [24] mit niedrig (ISCED 0–3), mittel (ISCED 3–4) und hoch (ISCED 5–8)), Hilfsmitteln (adaptiert nach Baker et al. [3]) und Zusammenleben (dichotomisiert: Ja = 1, Nein = 0) wurden folgende Variablen erhoben:

*Außerhäusliche Mobilität*: Aggregierte Variablen der außerhäuslichen Mobilität basieren auf den GPS-Rohdaten, die außerhalb des eigenen Zuhauses (50 m Pufferzone) erhoben wurden. Ein Stopp-und-Trip-Klassifikator zur Identifikation von Zeiten des Verweilens an einem Ort (Stopp) und der Bewegung durch außerhäusliche Umwelten (Trip) wurde während der Studie entwickelt und optimiert [25, 26] und dient zur Aggregation der GPS-Rohdaten. Das Rahmenkonzept von Fillekes et al. [27] dient seit 2019 als Grundlage für das standardisierte Berichten von GPS-bezogenen Variablen. Für vorliegende Arbeit wurden folgende 2 gängige GPS-Variablen aus diesem Rahmenkonzept übernommen, welche die zeitliche sowie die räumliche Facette außerhäuslicher Mobilität beschreiben:

- *Time out of Home (TOH):* kumulierte Zeit in Minuten pro Tag, die außerhalb des eigenen Zuhauses verbracht wurde. Als Tag wird hierbei die Zeitspanne von 3:00 Uhr morgens bis 2:59 Uhr des folgenden Tages gezählt, um Aktivitäten, die sich bis nach Mitternacht erstreckten, zu dem vorherigen Tag zählen zu können. Als gültige Tage gingen hierbei alle Tage ein, an denen mindestens 8 h GPS-Datenpunkte aufgezeichnet wurden [27].
- *Convex Hull (CHull):* Die „konvexe Hülle“ umfasst alle GPS-Datenpunkte eines Tages dargestellt als Polygon und wird als Maß eingesetzt, um die räumliche Facette der außerhäuslichen Mobilität zu beschreiben [27]. Die Maßeinheit ist hierbei km^2^ und kann auf einer Karte veranschaulicht werden (Abb. 1).

*Gesundheitskompetenz*: Zur Messung der Gesundheitskompetenz wurde die 16-Items umfassende Kurzversion [28] des HLS-EU‑Q (Health Literacy Survey Questionnaire) verwendet und gemäß den Vorgaben in einen Index (0–50) umgewandelt [29]. Höhere Werte bezeichnen eine höhere Gesundheitskompetenz.

*Gesundheitsbezogene Lebensqualität*: Zur Erhebung der gesundheitsbezogenen Lebensqualität wurde der SF-12 (Short Form Health Survey; [30]) eingesetzt, eine validierte Kurzform des SF-36 [31]. Hier können 2 Subskalen gebildet werden. Die körperliche Summenskala (KSK-12) erfasst die auf die körperliche Funktionsfähigkeit bezogene Lebensqualität, die psychische Summenskala (PSK-12) bezieht sich auf deren psychische Bereiche. Ausgewertet werden die Skalen in standardisierter Form und es können jeweils Werte zwischen 0 und 100 erreicht werden, wobei höhere Werte für eine höhere gesundheitsbezogene Lebensqualität stehen [31].

*Umweltbezogene Variablen****:*** Die umweltbezogenen Variablen Kreuzungsdichte, Anteil an Grünflächen und ÖPNV-Anbindung basieren auf einer Umkreis- und Näheanalyse der Bewegungsdaten und der geografischen Position des Zuhauses. Als Grundlage dienten die GPS-Koordinaten der Teilnehmenden sowie die frei zugängliche Plattform OpenStreetMap© (FOSSGIS e.V., Berlin, Deutschland), welche relevante Ortspunkte (z. B. Apotheken, Einkaufsmöglichkeiten oder Grünflächen) und Flächenkategorisierungen zur Verfügung stellt.

Zur Berechnung der *Kreuzungsdichte* als Maß der Fußgängerfreundlichkeit wurde jeweils ein 1500 m-Radius um das Zuhause gezogen und die Anzahl der Kreuzungen mit mindestens 3 Straßen gezählt, welche zu Fuß begangen werden können [32].

Für den *Anteil an Grünflächen* diente ein 500 m-Radius. Die Attribute, welche eine Grünfläche identifizieren (z. B. Garten, Park, Wald, Erholungsflächen) wurden gezählt, um die Proportion von Grünflächen zu sonstiger Fläche zu berechnen [32].

Für die *ÖPNV-Anbindung *wurde ein „begehbarer“ Radius gewählt, den die Teilnehmenden in 15 min (gemittelte Gehgeschwindigkeit der Teilnehmenden) zu Fuß erreichen können. Es wurden Attribute gezählt, die einen ÖPNV-Zugang kennzeichnen (z. B. Bushaltestelle oder Bahnhof).

Zusätzlich wurde die Umweltvariable *Gehwegqualität* mit dem Item: „Wie ist die Gehwegqualität unmittelbar vor der Wohnung?“, erhoben, welches vom Studienpersonal eingeschätzt wurde. Antwortmöglichkeiten waren „sehr schlecht“, „eher schlecht“, „eher gut“, „sehr gut“ (0–3); der daraus gebildete Mittelwert ging in die Analysen ein.

*Versorgung*: Für die Versorgung im ländlichen Raum wurden 3 Variablen gemessen: Inanspruchnahme von ambulanten Versorgungseinrichtungen, die Anzahl an Gesundheitseinrichtungen sowie die subjektive Erreichbarkeit ambulanter Versorgungseinrichtungen.

Die *Inanspruchnahme ambulanter Versorgungsleistungen *wurde mit dem eigenen Item: „Wie häufig haben Sie in den letzten drei Monaten eine der folgenden medizinischen Leistungen in Anspruch genommen?“ (Hausärztin/Hausarzt, Fachärztin/Facharzt, Physiotherapie, ambulanter Pflegedienst, Logopädie oder Ergotherapie, Psychotherapie, Sonstiges), abgefragt und zusammengefasst. Dabei wurde die Antwort „nie“ mit 0 kodiert, „1 im Monat“ mit 1, „1 Mal die Woche“ mit 2, „häufiger“ mit 3. In die Analysen ging der Mittelwert ein.

Für die *Anzahl an Gesundheitsrichtungen* wurde ein „begehbarer“ Radius gewählt, welchen die Teilnehmenden in 15 min (gemittelte Gehgeschwindigkeit der Teilnehmenden) zu Fuß erreichen können und Attribute, welche Gesundheitseinrichtungen identifizieren (z. B. Apotheke, hausärztliche Praxis, Krankenhaus), wurden gezählt.

Die *subjektive Erreichbarkeit von ambulanten Versorgungseinrichtungen *wurde mit 2 folgenden Fragen erhoben: „Wie einfach ist es für Sie, zu Ihrer Hausärztin oder Ihrem Hausarzt zu kommen?“ „Wie einfach ist es für Sie zur nächsten Bushaltestelle zu kommen?“ Dabei wurden die Antworten wie folgt codiert: 0 = sehr schwierig, 1 = schwierig, 2 = einfach, 3 = sehr einfach und der Mittelwert ging in die Analyse ein.

*Soziale Umwelt*: Die *Zufriedenheit mit dem sozialen Netzwerk* wurde mit dem Item: „Wie zufrieden sind Sie insgesamt mit Ihrer Beziehung zu den Personen, über die wir eben gesprochen haben?“ (0–10), erfragt. Dieses Item stammt aus dem Fragebogen zum sozialen Netzwerk aus dem „Survey of Health, Ageing and Retirement in Europe“ (SHARE; [33]).

*Aktives Autofahren*: Für dieses Item wurde gefragt, ob die Teilnehmenden einen gültigen Führerschein (PKW) besitzen und diesen in den letzten 4 Wochen aktiv genutzt haben.

**Abb. 2:**
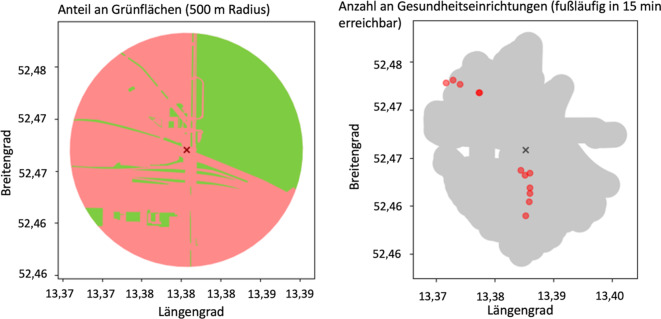
Exemplarische Darstellung von 2 objektiven, umweltbezogenen Variablen aus der MOBILE-Studie (*N* = 212) an einem fiktiven Beispiel. Das Kreuz in der Mitte repräsentiert den Wohnort eines Teilnehmenden und die Variablen werden individuell bestimmt. Für die Berechnung des Anteils an Grünflächen diente ein 500 m-Radius, für die Anzahl an Gesundheitseinrichtungen wurden alle relevanten Orte gezählt, die innerhalb von 15 min fußläufig erreichbar sind (Durchschnittsgeschwindigkeit der Teilnehmenden). Die *graue* Fläche zeigt den potenziell fußläufig begehbaren Raum. Die frei zugängliche OpenStreetMap© sowie die GPS-Daten der Teilnehmenden dienten als Grundlage der Berechnung. *GPS* Global Positioning System. (Quelle: eigene Abbildung)

Abb. 2 veranschaulicht exemplarisch die Berechnung von 2 Variablen (Anteil an Grünflächen und Anzahl an Gesundheitseinrichtungen), welche auf der Nähe- und Umkreisanalyse basieren.

### Statistische Analysen

Deskriptive Daten wurden für kategoriale Daten in absoluten und relativen Häufigkeiten ausgegeben und für kontinuierliche Variablen der Mittelwert und die Standardabweichung (SD). Alle Variablen wurden zentriert („grand mean centering“) und auf Normalverteilung überprüft. Dichotome Variablen wurden mit 0 und 1 codiert. TOH und CHull wurden als Zählvariable identifiziert und daher wurden generalisierte lineare gemischte Modelle (GLMM) unter der Annahme einer Poisson-Verteilung mit der außerhäuslichen Mobilität (TOH, CHull) als abhängige Variable berechnet. Messzeitpunkte auf Tagesebene (Level 1) wurden dabei in die Personenebene (Level 2) eingebettet. Alle Variablen und Kovariaten wurden in die Modelle eingefügt und um einen Zähler (Counter), welcher die einzelnen Messzeitpunkte (bis zu 7 pro Person) misst, ergänzt. Die Modelle wurden mittels der Maximum-Likelihood-Methode (Laplace Approximation) geschätzt. Ein Signifikanzlevel von 0,05 und ein Konfidenzintervall (KI) von 95 % wurden angenommen. Alle statistischen Analysen wurden mit R Version 4.3.0 (R Foundation for Statistical Computing, Wien, Österreich) und SPSS Version 29 (IBM Corporation, Armonk, New York, USA) für Windows (Microsoft Corporation, Redmond, Washington, USA) durchgeführt.

## Ergebnisse

Es nahmen 212 Personen mit einem Altersdurchschnitt von 81,5 Jahren an der MOBILE-Studie teil (SD: 4,1). Davon waren 119 (56,1 %) weiblich. Jeweils mehr als die Hälfte (50,7 %) wiesen eine hohe Bildung auf und lebten mit einer Person zusammen (57,1 %). Aktives Autofahren gaben 88,2 % an (97,8 % der Männer und 80,7 % der Frauen). Alle deskriptiven Ergebnisse sind in Tab. 1 dargestellt.

**Tab. 1:** Deskriptive Beschreibung ausgewählter soziodemographischer Merkmale der Teilnehmenden der MOBILE-Studie (*N* = 212)

Merkmale	M (SD)/*n* (%)
Alter, M (SD)	81,5 (4,1)
Geschlecht weiblich, *n* (%)	119 (56,1)
*Bildung, n (%)*	
Niedrig	5 (2,7)
Mittel	85 (40,1)
Hoch	122 (57,5)
*Wohnsituation, n (%)*	
Wohnt alleine	85 (40,1)
Mit Partner/in	121 (57,1)
Mit Familie	4 (1,9)
Sonstiges	2 (0,9)
*Hilfsmittel, n (%)*	
Hörhilfe	75 (35,4)
Sehhilfe	208 (98,1)
Gehhilfe	52 (24,5)
Internetzugang	154 (72,6)
Smartphone	183 (86,3)
Aktives Autofahren	187 (88,2)^a^
*Gesundheitskompetenz (HLS-EU-Q16), M (SD)*	
Gesamt-Index	40,6 (5,8)
*Gesundheitsbezogene Lebensqualität (SF-12), M (SD)*	
KSK-12	43,9 (11,3)
PSK-12	58,4 (4,9)
*Außerhäusliche Mobilität (GPS-basiert), M (SD)*	
TOH (in min)	319,3 (196,3)
CHull (in m^2^)	42,3 (132,8)
*Umweltbezogene Variablen (GIS-basiert; n* *=* *200), M (SD)*	
Kreuzungsdichte, 1500 m-Radius	92,9 (56,9)
Anteil an Grünflächen, 500 m-Radius	20,3 (15,4)
Gehwegqualität	1,8 (1,3)
ÖPNV-Anbindung in 15 min erreichbar^b^	3,7 (2,4)
*Versorgung, M (SD)*	
Inanspruchnahme ambulanter Versorgungsleistungen	2,8 (1,6)
Anzahl an Gesundheitseinrichtungen in 15 min erreichbar^2^	2,0 (4,6)
Subjektive Erreichbarkeit ambulanter Versorgungseinrichtungen	2,6 (0,7)
*Soziale Umwelt, M (SD)*	
Zufriedenheit mit dem sozialen Netzwerk	9,6 (1,0)

*CHull* Convex Hull, *GIS* Geographic Information System, *GPS* Global Positioning System, *HLS-EU-Q16* Health Literacy Survey, Kurzform mit 16 Items, *ISCED* International Standard Classification of Education, *KSK-12* körperliche Summenskala, *M* Mittelwert, *ÖPNV* Öffentlicher Personennahverkehr, *PSK-12* psychische Summenskala, *SD* Standardabweichung, *SF-12* Short Form Health Survey, *TOH* Time out of Home

^a^Davon 97,8 % der Männer und 80,7 % der Frauen

^b^Gemessen an der Durchschnittsgeschwindigkeit der Stichprobe

Die Ergebnisse der GLMM zeigen signifikant assoziierte Faktoren mit der außerhäuslichen Mobilität (Tab. 2). In Bezug auf die TOH zeigten das Alter (ß = −0,039, SE = 0,010, *p* < 0,001), die Zufriedenheit mit dem sozialen Netzwerk (ß = 0,123, SE = 0,005, *p* < 0,001), der Anteil an Grünflächen (ß = 0,005, SE = 0,003, *p* = 0,047), die Inanspruchnahme ambulanter Versorgungsleistungen (ß = 0,637, SE = 0,180, *p* < 0,001) und aktives Autofahren (ß = −0,361, SE = 0,127, *p* = 0,004) signifikante Assoziationen, während Geschlecht, Zusammenleben, KSK-12, PSK-12, Gesundheitskompetenz, Anzahl an Gesundheitseinrichtungen, ÖPNV-Anbindung, subjektive Erreichbarkeit ambulanter Gesundheitseinrichtungen, Kreuzungsdichte, Gehwegqualität und Bildung nicht signifikant assoziiert waren (*p* > 0,05). Bezüglich der CHull wiesen Zusammenleben mit einer anderen Person (ß = 0,689, SE = 0,328, *p* = 0,035), Gesundheitskompetenz (ß = 0,077, SE = 0,029, *p* = 0,08), Gehwegqualität (0,366, SE = 0,121, *p* = 0,003) und die Inanspruchnahme ambulanter Versorgungsleistungen (ß = 1,532 SE = 0,682, *p* = 0,027) signifikante Assoziationen auf, während Geschlecht, Alter, Zufriedenheit mit dem sozialen Netzwerk, KSK-12, PSK-12, Anzahl an Gesundheitseinrichtungen, ÖPNV-Anbindung, subjektive Erreichbarkeit ambulanter Versorgungseinrichtungen, Kreuzungsdichte, Anteil an Grünflächen, aktives Autofahren und Bildung nicht signifikant assoziiert waren (alle *p* > 0,05).

**Tab. 2:** Prädiktoren für außerhäusliche Mobilität. Ergebnisse der gemischten Modelle (MOBILE-Studie, *N* = 212)

	Modell 1 (TOH)				Modell 2 (CHull)			
Prädiktoren	β	SE	z Value	*p*	β	SE	z Value	*p*
(Achsenabschnitt)	5,860	0,377	15,542	**<0,001**	−3,025	1,413	−2,084	**0,037**
Geschlecht (Ref.: männlich)	−0,081	0,087	−0,928	0,353	−0,586	0,328	−1,784	0,074
Alter	−0,039	0,010	−3,750	**<0,001**	−0,072	0,040	−1,825	0,068
Zufriedenheit mit dem sozialen Netzwerk	0,123	0,005	24,695	**<0,001**	−0,024	0,034	−0,712	0,477
Zusammenleben (Ref.: Nein)	−0,075	0,087	−0,864	0,388	0,689	0,328	2,103	**0,035**
KSK-12	0,007	0,004	1,668	0,095	−0,001	0,160	−0,076	0,940
PSK-12	−0,010	0,008	−1,288	0,198	−0,040	0,030	−1,329	0,184
Gesundheitskompetenz	0,000	0,007	0,016	0,987	0,077	0,029	2,663	**0,008**
Anzahl Gesundheitseinrichtungen	0,015	0,009	1,670	0,095	−0,037	0,034	−1,076	0,282
ÖPNV-Anbindung	0,003	0,000	1,345	0,179	−0,005	0,003	−1,549	0,121
Subjektive Erreichbarkeit ambulanter Versorgungseinrichtungen	0,004	0,021	0,166	0,868	0,069	0,081	0,847	0,397
Kreuzungsdichte	0,004	0,589	0,075	0,940	0,269	0,225	1,195	0,232
Gehwegqualität	0,035	0,320	1,108	0,268	0,366	0,121	3,018	**0,003**
Anteil an Grünflächen	0,005	0,003	1,988	**0,047**	0,009	0,010	0,910	0,363
Inanspruchnahme ambulanter Versorgungsleistungen	0,637	0,180	3,532	**<0,001**	1,532	0,682	2,248	**0,025**
Führerscheinbesitz (Ref.: Nein)	−0,361	0,127	−2,845	**0,004**	0,232	0,488	0,475	0,635
Mittlere Bildung (Ref.: niedrig)	0,215	0,244	0,377	0,378	1,730	0,990	1,748	0,081
Hohe Bildung (Ref.: niedrig)	0,135	0,239	0,570	0,571	1,675	0,972	1,7234	0,085
Counter	0,067	0,001	78,159	**<0,001**	0,020	0,002	9,263	**<0,001**
*Random Effects (zufällige Effekte)*								
σ^2^	0,00				0,13			
τ_00_	0,25 _pseudonym_				3,44 _pseudonym_			
ICC	0,99				0,96			
*N*	194 _pseudonym_				194 _pseudonym_			
Beobachtungen	1261				1255			
Marginales R^2^/konditionales R^2^	0,296/0,990				0,282/0,974			

Fett markierte *p*-Werte stehen für ein Signifikanzniveau <0,05

*ß* Koeffizient, *SE* Standard Error, *Ref.* Referenzkategorie, *TOH* Time out of Home (Zeit außerhalb des Zuhauses), *CHull* Convex Hull (konvexe Hülle), *KSK-12* körperliche Summenskala des SF-12 (Short Form Health Survey), *PSK-12* psychische Summenskala des SF-12, *ÖPNV* Öffentlicher Personennahverkehr, *ICC* Interklassen-Korrelation,* R*^*2*^ Determinationskoeffizient

## Diskussion

Im Rahmen dieses Artikels wurden Assoziationen von persönlichen, sozialen und umweltbezogenen Faktoren sowie Versorgungsparameter auf die außerhäusliche Mobilität bei Menschen ab 75 Jahren im ländlichen Raum untersucht. Es zeigten sich positiv signifikante Assoziationen unterschiedlicher Facetten außerhäuslicher Mobilität mit zunehmendem Alter (TOH), höherer Zufriedenheit mit dem sozialen Netzwerk (TOH), Zusammenleben (CHull), höherer Gesundheitskompetenz (CHull), besserer Gehwegqualität (CHull), größerem Anteil an Grünflächen (TOH), einer höheren Inanspruchnahme ambulanter Versorgungsleistungen (TOH, CHull) und aktivem Autofahren (TOH). Die Teilnehmenden der MOBILE-Studie zeigten mit durchschnittlich über 300 min TOH und einer CHull von 42,3 km^2^ ein höheres [7, 34] Mobilitätsverhalten als vergleichbare Studien mit älteren, zu Hause lebenden Personen.

In der vorliegenden Studie zeigte sich ein Alterseffekt mit einer negativen Assoziation bei höherem Alter bezüglich außer Haus verbrachter Zeit. Ein Befund, der in zahlreichen Studien bestätigt wird (z. B. [35, 36]). In unserer Analyse zeigte sich kein Geschlechtereffekt und in der Literatur gibt es diesbezüglich gemischte Studienergebnisse. Akinci et al. und Fristedt et al. zeigten beispielsweise in ihren Studien eine positive Assoziation von männlichem Geschlecht und außerhäuslicher Mobilität [12, 37], während Giannouli et al. keinen Geschlechterunterschied ausmachen konnten [7]. Diese heterogenen Ergebnisse unterstreichen eine Studie von Matsuda et al., welche in Mediationsanalysen Wirkpfade untersucht haben und darstellten, dass dieselben Faktoren auf unterschiedliche Weise auf die Geschlechter und assoziierte außerhäusliche Mobilität wirken [38].

Die Hypothese 1 kann nur in Teilen bestätigt werden. So zeigten sich die körperliche und psychische Gesundheit nicht signifikant mit außerhäuslicher Mobilität assoziiert. Dies kann an der Limitation des Messinstrumentes auf die rein gesundheitsbezogene Lebensqualität liegen. So zeigten Rantakokko et al., welche ein breiter gefasstes Messinstrument zur Lebensqualität (WHOQOL-BREF) verwendeten, dass eine reduzierte Lebensqualität mit einer reduzierten außerhäuslichen Mobilität assoziiert ist [39]. Auch die Bildung zeigte sich nicht signifikant, was vor allem an dem sehr hohen Bildungsniveau in vorliegender Stichprobe liegen kann. So wiesen knapp 60 % ein hohes Bildungsniveau auf, ein Wert, der deutlich über dem der deutschen Allgemeinbevölkerung liegt (im Vergleich: 18 % der Altersgruppe 75+ mit höchstem Bildungsniveau nach Statistischem Bundesamt; [40]). Die Gesundheitskompetenz zeigte sich positiv signifikant assoziiert mit außerhäuslicher Mobilität, was andere Studien bestätigen [10, 41]. Die Interventionsstudie von Uemura et al. zur Förderung der Gesundheitskompetenz zeigte eine bedeutende Steigerung der Gesundheitskompetenz und eine signifikante Steigerung der außerhäuslichen Mobilität in der Interventionsgruppe – eine wichtige Erkenntnis, die zeigt, dass die Förderung von Mobilität auch sekundär über die Förderung anderer Faktoren gelingen kann. Auch das aktive Autofahren zeigte sich als persönliche Kompetenz mit einer positiven signifikanten Assoziation bezüglich außerhäuslicher Mobilität. So zeigten Personen, die aktiv Auto fahren eine größere Zeit an außerhalb des Zuhauses verbrachter Zeit, was sich durch die bessere Erreichbarkeit von Orten des täglichen Lebens – insbesondere im Ländlichen – erklären lässt. In vorliegender Stichprobe gaben fast alle Männer (97,8 %) an, dass sie aktiv Auto fahren, während es bei den Frauen knapp 80 % waren. Dieses Verhältnis deckt sich mit dem Fahrerlaubnisbestand [42] und verdeutlicht die geschlechtliche Ungleichheit bezüglich des Autofahrens im Alter. Die Bedeutung vom aktiven Autofahren im Alter ist in diversen Studien untersucht worden und so zeigten z. B. Hajek et al., dass aktives Autofahren auch im sehr hohen Alter mit verschiedenen Facetten von gesundheitsbezogener Lebensqualität assoziiert ist [43], und Chihuri et al. zeigten in einer Übersichtsarbeit, dass die Beendigung aktiven Autofahrens („driving cessation“) mit negativen Gesundheitsergebnissen wie Depression assoziiert ist [44].

Hypothese 2 untersuchte den Zusammenhang mit der sozialphysischen Umwelt und kann in Teilen bestätigt werden. So zeigt sich in der vorliegenden Studie eine positive signifikante Assoziation der außerhäuslichen Mobilität mit der Zufriedenheit in Hinblick auf das soziale Netzwerk und das Zusammenleben mit einer anderen Person. Dies hebt die Bedeutung von sozialen Aktivitäten im Alter hervor. Studien zeigten, dass sowohl das soziale Netzwerk der Freunde [10] als auch soziale Partizipation [9] positiv mit außerhäuslicher Mobilität korrelieren und als modifizierende Faktoren in Interventionsstudien eingebunden werden können. Weiterhin zeigte sich in vorliegender Arbeit, dass der Anteil an Grünflächen und die Gehwegqualität positiv mit außerhäuslicher Mobilität assoziiert sind. Hervorzuheben ist, dass es bisher wenig Forschung zu Umweltfaktoren und außerhäuslicher Mobilität im ländlichen Raum gibt und bisherige Forschungsergebnisse meist auf den urbanen Raum bezogen sind. Akinci et al. untersuchten beispielsweise Nachbarschaftsgefüge in Barcelona und zeigten positive Assoziationen mit dem Anteil an Grünflächen sowie dem Vorhandensein von Bänken [12]. Und eine systematische Übersichtsarbeit stellte fest, dass eine höhere Kreuzungsdichte positiv mit außerhäuslicher Mobilität assoziiert ist [45]. In vorliegender Arbeit war die Kreuzungsdichte jedoch nicht bedeutsam mit außerhäuslicher Mobilität assoziiert, was damit erklärt werden kann, dass die Studienregion, das Havelland in Brandenburg, sehr weitläufig ist und viele Strecken zwangsläufig mit dem Auto zurückgelegt werden, sodass die Fußmobilität mitunter eine untergeordnete Rolle spielt. Auch die ÖPNV-Anbindung war nicht signifikant assoziiert, was unter anderem an einer mangelnden ÖPNV-Infrastruktur liegen kann und anderseits daran, dass dem Autofahren auf dem Land einer höheren Bedeutung beigemessen wird. Hier sollte weitere Forschung folgen, da klimafreundlichere Alternativen zur Automobilität wichtiger werden, der Bedarf älterer Menschen an guter und verlässlicher ÖPNV-Infrastruktur steigen wird und positive Assoziationen unter anderem mit dem subjektiven Wohlbefinden gefunden wurden [46, 47].

Hypothese 3 untersuchte den Zusammenhang von ambulanter Versorgung und außerhäuslicher Mobilität, ein Forschungsgegenstand, der bisher kaum untersucht wurde. Hypothese 3 kann anteilig bestätigt werden. Die Inanspruchnahme ambulanter Gesundheitsversorgung zeigte in vorliegender Studie eine positive signifikante Beziehung mit außerhäuslicher Mobilität bezogen auf sowohl die zeitliche als auch die räumliche Facette. Hier könnte die Beziehung in beide Richtungen betrachtet werden. Einerseits erhöht eine größere Inanspruchnahme ambulanter Versorgungsleistungen, welche – insbesondere im ländlichen Raum – mit weiten Wegen und hohem Zeitaufwand verbunden sein kann, die außerhäusliche Mobilität, anderseits kann eine hohe außerhäusliche Mobilität auch als Ressource gesehen werden, solche Versorgungsleistungen überhaupt in Anspruch nehmen zu können. Erste Hinweise dazu wurden in einer Studie mit vulnerablen älteren Menschen mit funktionellen Einschränkungen und Depression gefunden, in der höhere außerhäusliche Mobilität positiv mit der Inanspruchnahme ambulanter hausärztlicher Versorgung und psychiatrischer Versorgung in der Praxis assoziiert war [11]. Das Vorhandensein sowie die subjektive Erreichbarkeit von Gesundheitseinrichtungen waren jedoch nicht signifikant mit einer Facette außerhäuslicher Mobilität assoziiert, was vermuten lässt, dass das Aufsuchen einer Gesundheitseinrichtung weniger mit dem Vorhandensein oder der Erreichbarkeit, sondern mehr mit einer bewussten Entscheidung assoziiert ist und weitere Wege in Kauf genommen werden.

### Stärken und Limitationen

Neben dem Einsatz objektiver Mobilitätsmessung und dem Fokus auf den ländlichen Raum, stellt die Ergänzung der GPS-Daten um objektiv gemessene Umweltfaktoren eine weitere Stärke dar. Auch das große Sample einer Gruppe mit vergleichsweise hohem Alter kann positiv hervorgehoben werden, da viele Alternsstudien sich mit dem jüngeren Alter befassen.

Eine Limitation ist, dass auch bei der objektiven Messung Ungenauigkeiten entstehen können. So kann das Ergebnis durch technische Probleme (Ausfälle der App, kein GPS-Empfang, Akku zu schnell entladen) oder durch Anwendungsprobleme (Mobiltelefon zu Hause vergessen, Akku nicht geladen) verfälscht sein. Weiterhin wurden bei einigen Variablen (Inanspruchnahme ambulanter Versorgungsleistungen, Zufriedenheit mit dem sozialen Netzwerk) einzelne Items verändert bzw. aus einer bestehenden Skala einzeln herausgenommen, was die Aussagekraft verringern kann. Wünschenswert wäre es gewesen, validierte Skalen zu verwenden, was in vorliegender Weise in dieser Arbeit aber nicht möglich war.

Weiterhin kann es bei den objektiven, ortsbezogenen Variablen zu Ungenauigkeiten kommen, falls die Attribute (des OpenStreetMap©-Projektes) nicht richtig vergeben wurden. Zudem beziehen sich die Ergebnisse in vorliegender Studie auf die Auswertung der Fragebögen und der GPS-Messung, bei der die außerhäusliche Mobilität in zeitlicher und räumlicher Facette beschreibt, aber nicht in der Fortbewegungsart differenzieren kann. Eine Auswertung der Bewegungstagebücher konnte ressourcenbedingt für dieses Arbeit nicht integriert werden, hätte aber eine größere Aussagekraft haben können, da dort Fortbewegungsarten abgefragt wurden. So kann keine Aussage beispielsweise zur Fahrradmobilität oder Fußmobilität gemacht werden, was aber beides eine hohe Relevanz hat. Bezüglich des ÖPNV wurde ausschließlich die Anbindung untersucht, eine differenzierte Betrachtung beispielsweise bezogen auf die ÖPNV-Nutzung hätte eine höhere Aussagekraft gehabt.

Abschließend lassen sich durch die vorliegenden Daten und Analysen keine kausalen Zusammenhänge untersuchen und es können nur Assoziationen berichtet werden.

## Fazit und Implikationen für die Forschung und Praxis

Es zeigte sich, dass einige bekannte Assoziationen, welche bisher vor allem im urbanen Raum untersucht wurden, auch für den ländlichen Raum zutreffen. So zeigen Umweltfaktoren wie eine gute Gehwegqualität und der Anteil an Grünflächen einen positiven Zusammenhang mit außerhäuslicher Mobilität und sind daher auch für die Planung der baulichen Umwelt auf dem Land wichtig. Eine Erkenntnis aus der vorliegenden Studie ist die signifikante Assoziation der Inanspruchnahme ambulanter Versorgungsleistungen. Andere Versorgungsvariablen wie die Verfügbarkeit und die subjektive Erreichbarkeit von Versorgungseinrichtungen waren hingegen nicht signifikant mit der außerhäuslichen Mobilität verknüpft, was die Bedeutung der Inanspruchnahme hervorhebt. Die Inanspruchnahme von ambulanten Versorgungseinrichtungen als Alltagsaktivität ist daher ein nicht zu unterschätzender Faktor, der die außerhäusliche Mobilität im Alter beeinflussen kann und auch in weiteren Mobilitätsstudien berücksichtigt werden sollte. Das aktive Autofahren stellt einen weiteren bedeutenden Befund aus vorliegender Studie dar. So war nicht nur der Anteil an aktiv Autofahrenden – insbesondere bei den Männern – sehr hoch, auch hebt die signifikante Assoziation zur außerhäuslichen Mobilität die Bedeutung der Automobilität auf dem Land hervor. Auch modifizierbare Faktoren, wie beispielsweise das soziale Netzwerk und die Gesundheitskompetenz, können eine wichtige Rolle beim Erhalt der außerhäuslichen Mobilität im Alter spielen und sollten stärker in die Forschung, vor allem im Rahmen von Interventionsstudien, eingebunden werden. Ländliche Räume, die selbst sehr heterogen sein können, haben dabei besondere Charakteristika, welche in Studien berücksichtigt werden sollten.
